# Mapping the research landscape of Mild Cognitive Impairment in Parkinson’s disease: a bibliometric and visualization analysis

**DOI:** 10.3389/fnagi.2025.1624420

**Published:** 2025-09-10

**Authors:** Yufeng Peng, Hao Chen, Kewei Peng, Luyao Li, Zihua Ma

**Affiliations:** ^1^Department of Neurology, Zhenhai Hospital of Traditional Chinese Medicine, Ningbo, China; ^2^Department of Rehabilitation, Wuhan No. 4 Hospital, Wuhan, China

**Keywords:** Parkinson’s disease, Mild Cognitive Impairment, bibliometric analysis, visualization analysis, research frontiers

## Abstract

**Background:**

Mild Cognitive Impairment in Parkinson’s disease, a common non-motor symptom of Parkinson’s disease, significantly impacts individuals’ quality of life and predicts dementia risk, underscoring its clinical research importance. This study aimed to characterize the global bibliometric landscape and identify research hotspots, knowledge gaps, and future trends in the PD-MCI field over the past two decades using bibliometric and visualization methods.

**Methods:**

Literature related to PD-MCI published between 2005 and 2024 was retrieved from the Web of Science Core Collection database. Tools such as CiteSpace and VOSviewer were employed for visual analysis of annual publication volume, country/institutional distribution, author collaborations, journal co-citations, and keyword co-occurrence and bursts, constructing knowledge maps.

**Results:**

Mild cognitive impairment in Parkinson’s disease research demonstrated significant growth, with sustained increases in annual publication volume and citation frequency. The United States dominated the field, while research output from countries like China grew rapidly. Research hotspots evolved from early explorations of molecular mechanisms toward clinical and translational studies focusing on neuroimaging, biomarkers, application of MDS diagnostic criteria, non-motor symptoms, and cognitive subtypes. Recently, machine learning, multi-omics integration, neuroinflammation, and mitochondrial function have emerged as new frontiers.

**Conclusion:**

Mild cognitive impairment in Parkinson’s disease research has progressed from basic mechanism exploration to a multidisciplinary, integrated clinical-basic stage, following an evolutionary path of “pathological mechanism - clinical phenotype - biomarker - intervention strategy.” Future research should focus on unifying diagnostic criteria, deepening understanding of multifactorial pathological mechanisms, developing precise biomarker combinations, and exploring individualized intervention strategies to achieve early warning and disease modification for PD-MCI.

## Introduction

Parkinson’s disease (PD), the second most prevalent neurodegenerative disorder globally, is characterized by clinical features extending beyond motor disturbances; non-motor symptoms, particularly cognitive dysfunction, have emerged as a central issue impacts individuals’ quality of life and disease prognosis (Monastero et al., 2018). In recent years, Mild Cognitive Impairment in Parkinson’s Disease (PD-MCI), representing a transitional state between normal cognition and dementia, has garnered considerable academic attention. Epidemiological data indicate that approximately 20%–50% of individuals with PD manifest PD-MCI in the early stages of the disease (Chaudhary et al., 2020; Guo et al., 2021), with up to 30% of cases presenting with significant cognitive impairment at the time of diagnosis. This suggests that PD-MCI is not only an independent predictor of dementia development but also serves as a critical marker for the therapeutic window (Guo et al., 2019). Currently, the most widely accepted diagnostic criteria for PD-MCI are those established by the International Parkinson and Movement Disorder Society (MDS) Task Force in 2012 (Litvan et al., 2012), which require a diagnosis of idiopathic Parkinson’s disease (PD), a gradual cognitive decline reported by the patient/informant or observed by the clinician, cognitive impairment on standardized neuropsychological testing involving at least two tests with performance, for example, ≥1.5 SD below normative data, and cognitive deficits that do not interfere significantly with functional independence. With the escalating global aging population, the early identification and management of PD-MCI are of profound significance for delaying disease progression and mitigating socioeconomic burden.

Current research underscores the marked heterogeneity of PD-MCI, with cognitive deficits spanning multiple domains, including executive function, attention, visuospatial abilities, and memory (Wallace et al., 2022). Neuropathological studies implicate synergistic interactions between a-synuclein pathology and contributions from several other neurotransmitter systems, including dopamine and perhaps especially cholinergic dysfunction in PD-MCI pathogenesis (Zhang et al., 2018; van der Zee et al., 2022; Real et al., 2023; Li et al., 2024; Yoo et al., 2024). Advances in imaging biomarkers reveal that alterations in white matter microstructure identified via diffusion magnetic resonance imaging (dMRI) and patterns of cortical metabolic abnormality can provide objective biomarkers for the early diagnosis of PD-MCI (Monchi et al., 2024; Silva-Rodríguez et al., 2025). Notably, interactions between vascular risk factors and motor subtypes may exacerbate cognitive impairment through mechanisms involving neuroinflammation and blood-brain barrier disruption (Malek et al., 2016; Athauda et al., 2022), thereby offering novel avenues for multi-targeted therapeutic strategies.

It is crucial to distinguish PD-MCI from the more commonly studied amnestic Mild Cognitive Impairment (aMCI), often considered a prodromal stage of Alzheimer’s disease (AD). While both represent transitional states to dementia, key differences exist. In contrast to aMCI, which primarily features prominent memory deficits reflecting medial temporal lobe and hippocampal pathology associated with amyloid-β and tau, PD-MCI typically exhibits a more heterogeneous cognitive profile (Arruda et al., 2023; Maggi et al., 2024). Deficits in executive function, attention, and visuospatial abilities may be more prominent than memory impairment in early PD-MCI; deficits may fronto-striatal circuit dysfunction, dopaminergic degeneration, α-synuclein pathology in multiple networks, as well as contributions from co-pathologies (Xu et al., 2021; Yang et al., 2023; Hjelle et al., 2025). There are guidelines and suggestions for neuro-psychological testing that is relevant for PD-MCI [e.g., (Bezdicek et al., 2025; Biundo et al., 2025)]. This distinct pathophysiology, clinical presentation, and progression trajectory underscore the necessity for dedicated research and specific bibliometric analysis focused solely on PD-MCI.

Despite significant progress in PD-MCI research, the field confronts three principal challenges: Firstly, the lack of complete unification in diagnostic criteria and cognitive assessment tools results in considerable heterogeneity across studies. Secondly, longitudinal data indicate that predictive models for the conversion of PD-MCI to dementia lack systematic integration of gene-environment interactions (Puig-Davi et al., 2024). Thirdly, although pharmacological interventions, such as cholinesterase inhibitors, have demonstrated partial efficacy in randomized controlled trials, their precise mechanisms of action on specific cognitive domains and their long-term safety profiles require further rigorous validation (Mamikonyan et al., 2015; Loprinzi et al., 2018). In this context, conducting a global bibliometric study can provide a panoramic overview of knowledge evolution, identify interdisciplinary collaboration networks, and reveal regional research characteristics and disparities in resource allocation, thereby furnishing a scientific basis for optimizing research paradigms and formulating individualized diagnostic and therapeutic guidelines. Therefore, the primary objectives of this bibliometric analysis were to characterize the global research landscape of PD-MCI over the past two decades, encompassing publication/citation trends, country/institutional contributions, collaborative networks, core journals, and knowledge structures; to identify evolving research hotspots and emerging frontiers through keyword and reference analysis; and to highlight current knowledge gaps and suggest potential future research directions based on the synthesized findings.

## Method

### Search strategy

**FIGURE 1:**
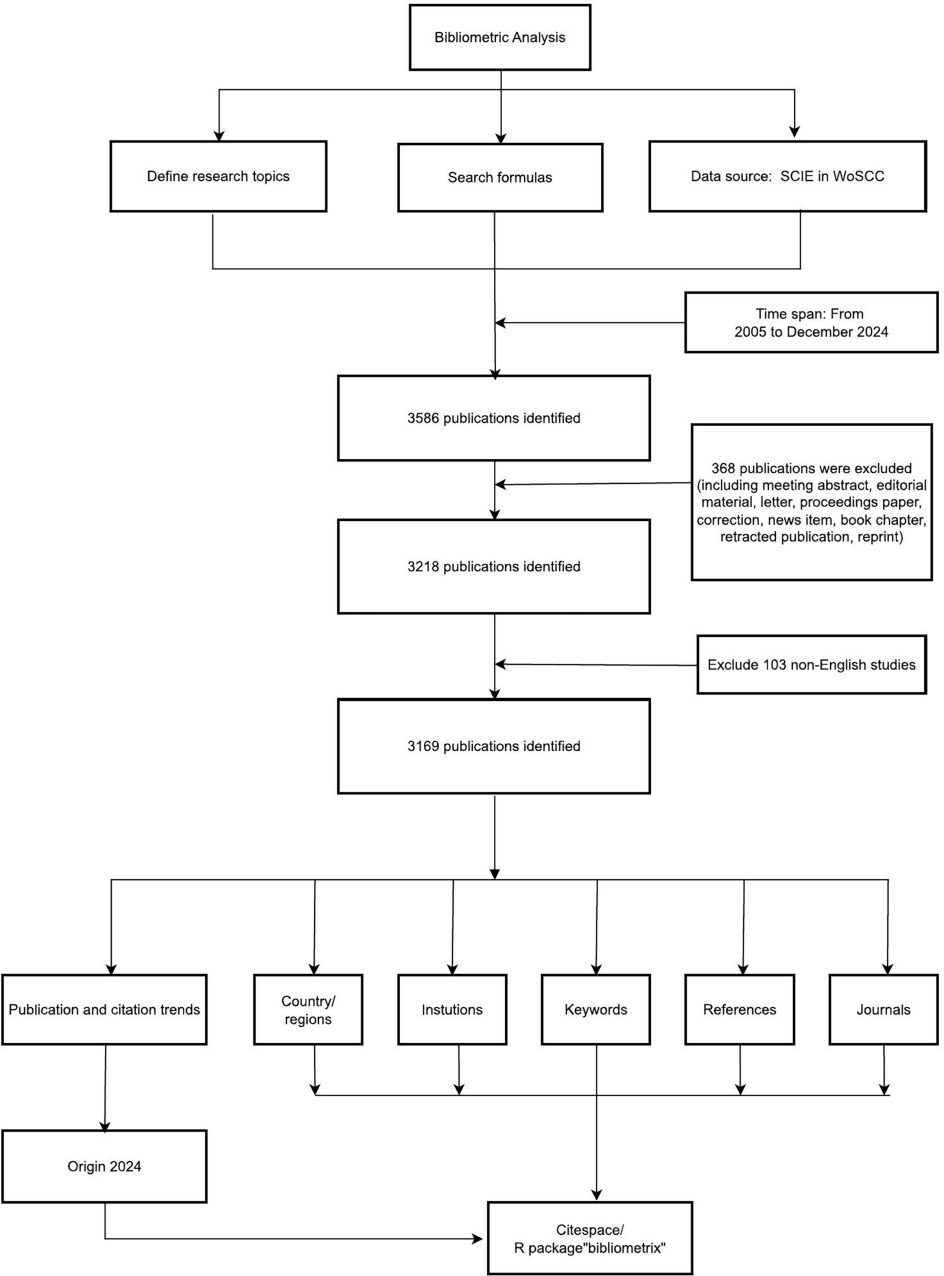
Flowchart of the study.

The Web of Science Core Collection (WoSCC), specifically its Science Citation Index Expanded (SCIE) subset, was selected as the primary data source for this bibliometric analysis. WoSCC is globally recognized as an authoritative citation database and is the predominant source used in bibliometric studies due to its comprehensive coverage of high-impact journals in the sciences, its robust citation indexing enabling network analysis, and the standardized metadata format essential for tools like CiteSpace and VOSviewer. While databases like SSCI, PsycINFO, and CINAHL cover valuable social science, psychology, and nursing/allied health literature, our primary focus was on capturing the core biomedical and clinical research output on PD-MCI, which is predominantly indexed in SCIE. Furthermore, using a single, well-structured database like WoSCC ensures data consistency and compatibility across the bibliometric software tools employed, which is critical for reproducibility. Expanding the search to multiple databases with differing indexing standards and export formats would introduce significant challenges in data deduplication, harmonization, and analysis workflow integration, potentially compromising the reliability and comparability of the bibliometric network analyses. This study collected literature pertaining to Mild Cognitive Impairment in Parkinson’s Disease published between January 1, 2005, and December 31, 2024. The retrieved dataset encompassed comprehensive citation metadata essential for knowledge graph analysis, including article titles, abstracts, keywords, author affiliations, institutional/country distributions, citation frequencies, and collaboration networks. Within the National Library of Medicine (NLM) database, we identified 11 terms using the search query “Parkinson disease” and 6 terms using “Mild cognitive impairment.” Based on the MeSH vocabulary, related entries, and our research objectives, we developed a comprehensive search strategy: “(((((((((((TS = (Idiopathic Parkinson Disease)) OR TS = (Idiopathic Parkinson’s Disease)) OR TS = (Lewy Body Parkinson Disease)) OR TS = (Lewy Body Parkinson’s Disease)) OR TS = (Paralysis Agitans)) OR TS = (Parkinson Disease, Idiopathic)) OR TS = (Parkinson’s Disease)) OR TS = (Parkinson’s Disease, Idiopathic)) OR TS = (Parkinson’s Disease, Lewy Body)) OR TS = (Primary Parkinsonism)) OR TS = (Parkinsonism, Primary) AND (((((TS = (Mild Cognitive Impairment)) OR TS = (Cognitive Impairment, Mild)) OR TS = (Cognitive Impairments, Mild)) OR TS = (Impairment, Mild Cognitive)) OR TS = (Impairments, Mild Cognitive)) OR TS = (Mild Cognitive Impairments)).” The remainder can be found in Supplementary Digital Content 1. Document types were restricted to Articles and Review Articles, while editorial materials, meeting abstracts, early access publications, notes, book chapters, letters, retracted publications, and corrections were excluded. Ultimately, following title and abstract screening, 417 irrelevant documents were excluded, resulting in the inclusion of 3,169 publications for analysis. The detailed process is illustrated in Figure 1.

### Data processing and analysis

Data processing and analysis involved extracting, cleaning, and standardizing the dataset from Web of Science Core Collection (WoSCC), which included “complete records and cited references” downloaded as plain text files. Raw data were directly imported into bibliometric software without format conversion, adhering strictly to PRISMA guidelines (Page et al., 2021). Following retrieval, two independent reviewers (Yufeng Peng and Kewei Peng) screened titles and abstracts, with exported data labeled as “download_xxx.txt” containing metadata such as titles, publication years, authors, affiliations, keywords, abstracts, and journal information. Three complementary bibliometric tools–VOSviewer 1.6.2, CiteSpace 6.1.R, and Bibliometrix 4.1–were employed to analyze 20 years of PD-MCI research. CiteSpace, developed by Chen (2004) enabled visualization of citation networks and emerging trends through progressive knowledge domain mapping. VOSviewer optimized large-scale data interpretation by constructing interpretable bibliometric maps, while Bibliometrix, an R-based platform, facilitated scientific mapping using the “bibliometrix” package (Aria et al., 2017). These tools synergistically identified high-impact publications, collaboration patterns, and conceptual clusters, ensuring rigorous analysis of temporal trends and knowledge structures.

### Visualization analysis

The multi-dimensional analytical framework encompassed author networks, national/institutional distributions, journal patterns, citation linkages, and keyword evolution. VOSviewer 1.6.2 facilitated co-occurrence and clustering analyses of institutions and authors, where node size reflected element prominence and color-coded clusters indicated conceptual groupings. CiteSpace 6.1.R enabled dual-map overlays of journal distributions, timeline visualization of citation bursts, and keyword co-occurrence clustering, with cluster labels derived from title terms, keywords, and abstract semantics of representative publications. Bibliometrix 4.1 generated temporal publication trends for institutions/authors and keyword heatmaps reflecting conceptual intensity. Journal impact factors were extracted from the 2024 Journal Citation Reports (JCR) in WoSCC, while SCImago Graphica 1.0.48 and Origin 2024 enhanced graphical representation and statistical validation of spatial-temporal patterns. This integrated approach systematically decoded collaborative networks, knowledge diffusion pathways, and disciplinary convergence characteristics across spatiotemporal dimensions.

## Results

### Annual publication volume, citation volume and trends

**FIGURE 2:**
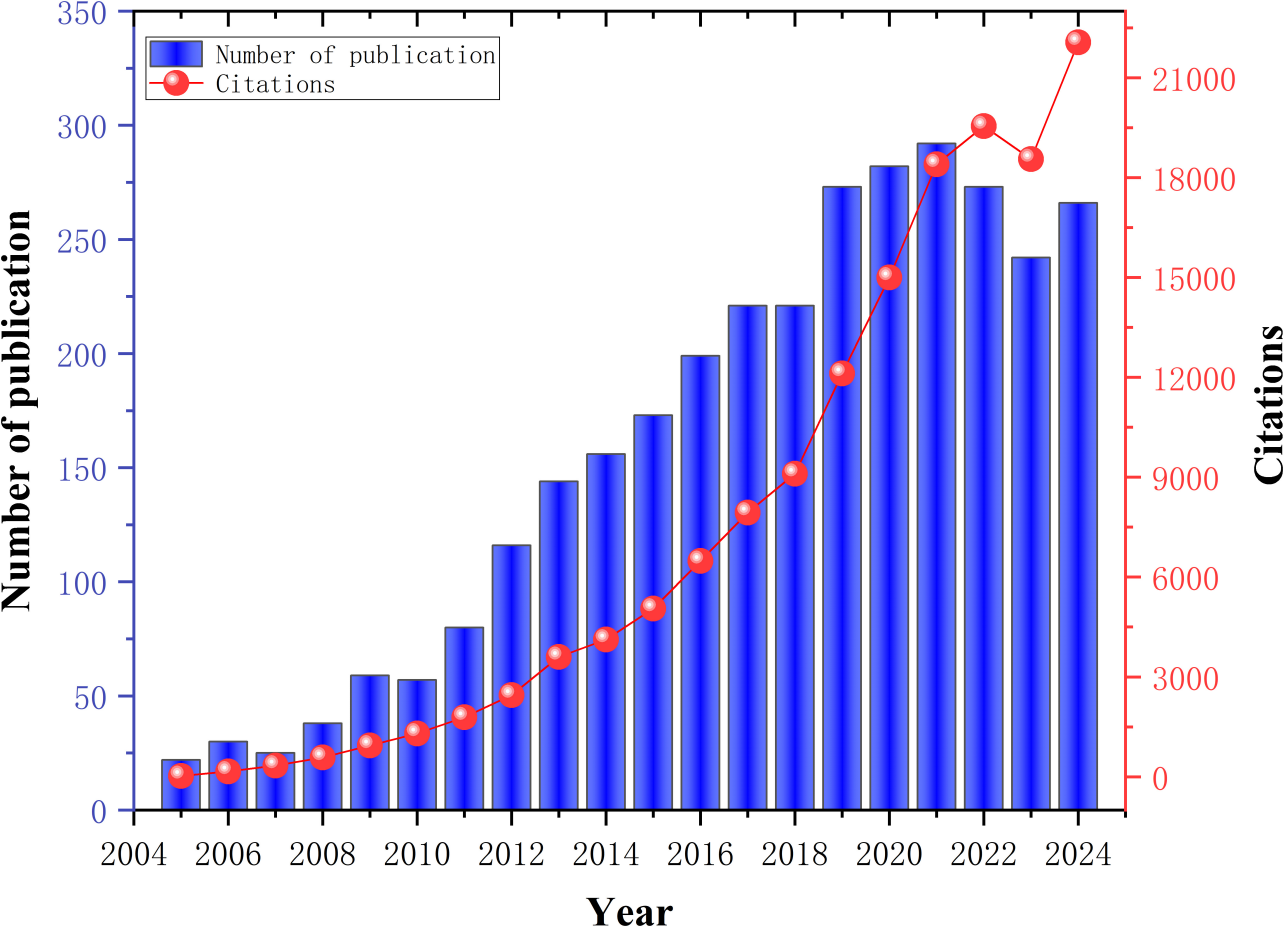
Trends in annual publications and cited articles on PD-MCI between 2005 and 2024.

A total of 3,169 publications were retrieved in this study. Figure 2 illustrates the annual and cumulative publication trends in PD-MCI from 2005 to 2024. Annual publication volume commenced at 22 articles in 2005, underwent sustained growth, surpassed 100 articles in 2012 and 200 articles in 2018, before reaching a peak of 292 articles in 2021. Despite a slight decline in the subsequent 2 years, publication volume remained substantial at 266 articles in 2024, representing an overall increase exceeding twelve-fold and reflecting sustained high research interest in this domain. Concurrently, the trajectory of annual citations demonstrated even more precipitous growth, escalating sharply from a mere 25 citations in 2005. This trend became particularly pronounced after 2015, exhibiting exponential growth to reach 22,062 citations by 2024. This surge indicates a dramatic expansion in the visibility, recognition, and academic impact of research in this field.

A strong positive correlation exists between publication volume and citation counts. Notably, particularly during the latter half of the study period, the growth rate of citations significantly outpaced that of publications. Even after publication volume peaked in 2021, annual citations continued to demonstrate substantial growth, suggesting that published research, especially recent literature, is progressively accumulating considerable academic influence and stimulating further investigation. Overall, research on PD-MCI has evolved from a relatively nascent field in the early 21st century into a highly active and influential research direction. The rapid proliferation of publications, and particularly the dramatic increase in citations, collectively underscore the escalating importance of PD-MCI in both clinical practice and scientific investigation. It has consequently emerged as a rapidly advancing and increasingly central topic within the broader landscape of neurodegenerative disease research.

### Country/region and institutional analysis

**FIGURE 3:**
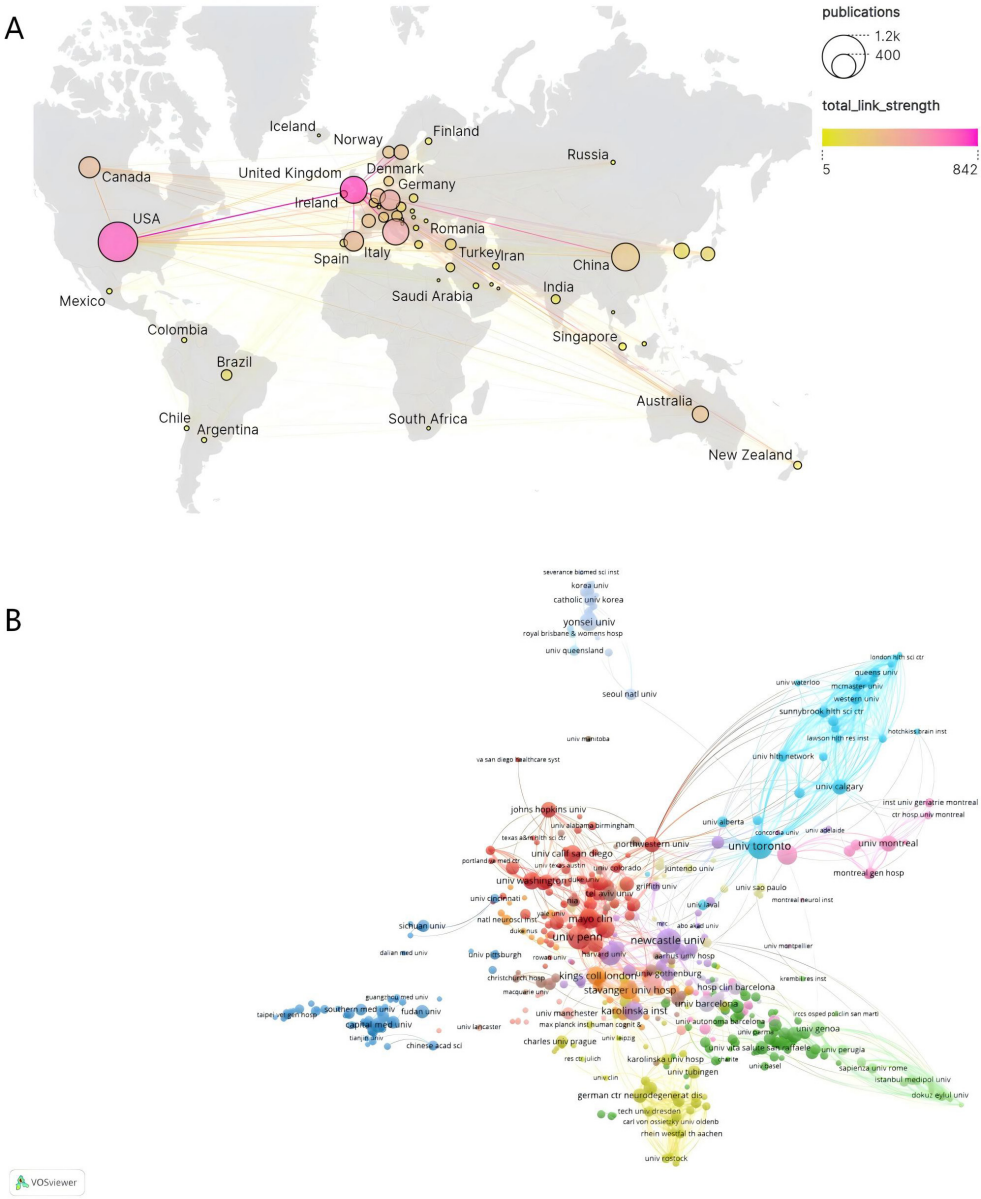
**(A)** Geographic visualization of country/area collaboration. **(B)** Co-analysis of the top 50 most productive institutions in the network visualization map.

A total of 3,169 publications involved contributions from 3,673 institutions across 83 countries/regions. As presented in Figure 3A and Table 1, the top 10 producing countries were ranked by publication volume. The United States (USA) demonstrated absolute leadership in this field, with its publication output (922 articles), total citation frequency (64,138 citations), and network centrality (0.32) being significantly preeminent. This indicates not only a substantial scale of research production and high impact but also a pivotal role as a central hub within the international collaboration network. Italy ranked second with 406 publications and, by virtue of its high total citation frequency (21,876 citations) and centrality second only to the USA (0.17), emerged as a significant research contributor and collaborative node. Although China ranked third with 404 publications, showcasing robust research productivity, its average citations per publication (39.82) and network centrality (0.05) were relatively lower, which may reflect factors such as the more recent rapid expansion of PD research output in China, potential differences in publication language focus, or variations in research collaboration patterns compared to established networks in the US and Europe. Notably, several countries, while not having the highest total publication volume, exhibited substantial research impact. Australia (average citations: 90.13), the Netherlands (88.48), Germany (84.49), and the United Kingdom (81.49) ranked among the highest in terms of average citations per publication, reflecting the high quality and impact of their research outputs. Among these, Australia (0.13) and the Netherlands (0.11) also demonstrated considerable network centrality. In contrast, South Korea, despite being among the top 10 producing countries, ranked relatively lower across various citation metrics and centrality (0.01). In summary, the global landscape of PD-MCI research is characterized by US leadership, with significant contributions from multiple European nations and Australia due to their high-quality research. China, as a high-output country, possesses considerable potential for enhancing its research impact and deepening international collaborations. Network analysis further confirmed that the USA and Italy are key centers for international collaboration in this field.

**TABLE 1:** Publications and citations in the top ten most productive countries/regions and institutions.

Country	Publications	Citations	Average citation	Centrality	Institutions	Publications	Citations	Average citation	Centrality
USA	**922**	**64138**	69.56	0.32	University of London	**138**	4964	35.97	0.03
Italy	406	21876	**53.88**	0.17	US Department of Veterans Affairs	131	**9404**	**71.79**	0.05
China	404	16088	39.82	0.05	Veterans Health Administration	130	**10308**	**79.29**	0.04
England	380	30967	**81.49**	0.08	University of California System	128	7164	55.97	0.05
Canada	262	16041	61.23	0.03	University of Toronto	103	**10889**	**105.72**	0.03
Germany	242	20446	**84.49**	0.09	Newcastle University	96	6252	65.13	0.04
Spain	229	16527	72.17	0.05	University of Pennsylvania	87	6157	70.77	0.05
Australia	157	14151	90.13	0.13	University College London	85	7877	**92.67**	0.03
South Korea	141	4112	29.16	0.01	Mayo Clinic	82	5401	65.87	0.1
Netherlands	130	11502	**88.48**	0.11	Helmholtz Association	80	1498	18.73	0.02

Values in bold indicate the highest or significantly higher values within the Top 10 for that metric.

From a geographical perspective, North America–especially the United States–demonstrates an overwhelming central position. Not only does it lead the world in research output, but it also radiates influence across Europe and Oceania through dense collaboration networks, forming a research hub characterized by high productivity and strong connectivity. Europe, by contrast, presents a multipolar collaborative ecosystem: major countries such as the United Kingdom and Germany sustain moderately strong cooperation through stable intra-regional linkages, yet their capacity for cross-regional collaboration remains limited. The Asia-Pacific region shows a structural imbalance between scale and effectiveness–China ranks among the top in publication volume but has not translated this into a corresponding role as an international collaboration hub, while Japan and South Korea tend toward a more inward-looking research model. Oceania, conversely, displays unusually high collaborative efficiency: countries such as Australia achieve highly effective global connections with medium-scale output, exemplifying an intensive cooperation strategy. South America and Africa remain largely at the periphery of the academic network, with individual countries sustaining only minimal participation through sporadic ties to European and American research chains. This hierarchical pattern highlights the pronounced regional inequality in resource integration and collaborative opportunities within the PD-MCI research system.

At the institutional level, University College London (138 publications), along with the US Department of Veterans Affairs (131 publications), the Veterans Health Administration (130 publications), and the University of California System (128 publications), constitute the core forces in research output within this domain. In terms of research impact, the University of Toronto distinguished itself with the highest total citation frequency (10,889 citations) and an exceptionally high average citation rate (105.72 citations per publication). University College London (92.67 citations per publication) also demonstrated strong performance, collectively representing high-impact research in the field alongside the US Veterans Health Administration (10,308 citations) and the Department of Veterans Affairs (9,404 citations). This landscape is visually represented in Figure 3B. This figure illustrates a complex global network of institutional relationships, where connections between nodes signify collaborative links. Institutions in the map are differentiated by color into multiple clusters, clearly indicating that collaboration is not uniformly distributed but rather forms collaborative groups centered around specific institutions or regions. This aligns with the characteristics revealed in Table 1, highlighting the predominance of North American and European institutions. Although most top-tier institutions exhibited generally low network centrality scores, suggesting that overall collaboration might be relatively dispersed or possess multiple centers, the map intuitively confirms the existence of close collaborative relationships, particularly within specific clusters. Notably, the Mayo Clinic, despite not ranking in the top five by publication volume (82 publications), demonstrated a network centrality score of 0.1, significantly higher than other leading institutions. This suggests it may play a particularly crucial bridging or hub role within the overall collaborative network structure, connecting different research clusters or directions. In conclusion, PD-MCI research is predominantly led by top universities and healthcare systems in the United Kingdom, the United States, and Canada. These institutions not only contribute a substantial volume of high-impact literature but also form collaborative networks that powerfully drive knowledge production and exchange in the field, clearly delineating a global collaboration landscape centered around North America and Europe.

### Authors and co-cited authors

**FIGURE 4:**
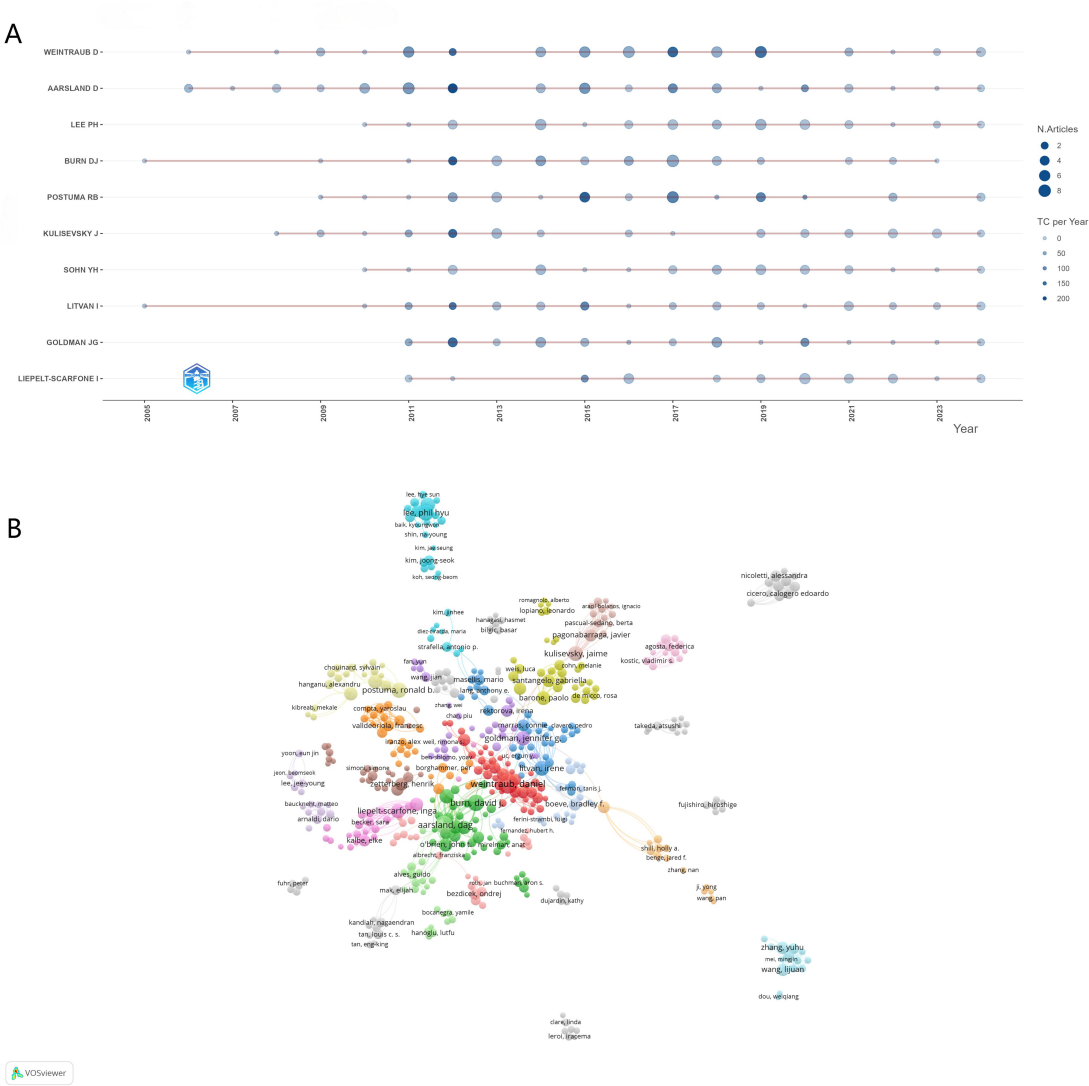
**(A)** Timeline maps of the number of articles and citations for the top 10 most productive authors. **(B)** Visualization of the top 100 most cited co-cited authors network.

This study identified 14,175 contributors to PD-MCI research. Figure 4A and Table 2 highlight the top 10 contributors. Weintraub, D. emerged as the most prolific author, having published 56 papers, followed by Aarsland, D. (45 papers) and Burn, D.J. (37 papers). Citation metrics reflect academic recognition, with 8 of the top 10 authors having garnered over 4,000 citations. The h-index, which synthesizes productivity and impact, further substantiates the significant contributions of these authors (Roldan-Valadez et al., 2019). Co-citation analysis revealed that Aarsland, D. was the most frequently co-cited author in PD-MCI research, indicating a foundational influence on the PD-MCI research paradigm. Figure 4B illustrates a closely interconnected co-authorship network among the top 100 cited authors, particularly clustered around highly productive researchers. These findings suggest that, over the two-decade period, collaborative networks have driven knowledge dissemination and methodological convergence, reflecting growing academic engagement and intellectual influence within PD-MCI research.

**TABLE 2:** Top 10 authors and co-cited authors related to PD-MCI.

Rank	Author	Count	Citations	H-index	Co-cited author	citations
1	Weintraub, d	**56**	**7908**	**38**	Aarsland, d	**2008**
2	Aarsland, d	45	**7654**	**40**	Litvan, i	1369
3	Burn, dj.	37	5047	31	Braak, h	980
4	Lee, ph	35	1084	20	Postuma, rb	921
5	Postuma, rb.	33	4534	29	Emre, m	914
6	Kulisevsky, j	31	4101	20	Williams-gray, ch	711
7	Litvan, i	31	4665	21	Petersen, rc	624
8	Barker, r.	29	5253	24	Dubois, b	613
9	Goldman, jg.	29	4373	25	Goetz, cg	589
10	Liepelt-scarfone, i	28	1314	13	Hughes, aj	588

Values in bold indicate the highest or significantly higher values within the Top 10 for that metric.

### Visualization analysis of journals and cited journals

**FIGURE 5:**
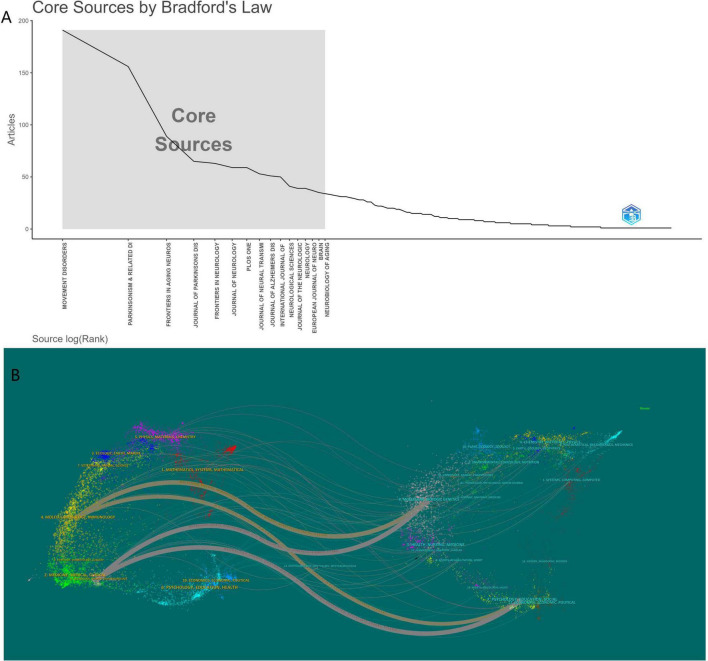
**(A)** Core sources by Bradford’s law. **(B)** Dual map coverage of journals of PD-MCI.

Between 2005 and 2024, 603 journals published articles related to PD-MCI. Bradford’s Law can identify core journals within a specific field (Venable et al., 2016). Figure 5A identified 16 core journals. Table 3 provides a detailed list of the top 10 journals ranked by the number of publications in this research area. Web of Science reported the 2023 Journal Citation Reports (JCR) and Impact Factors (IF). In terms of journal output, Movement Disorders was not only the journal publishing the most related articles (192 articles) but also the most cited journal (18,108 citations). Its high Impact Factor (7.4) and JCR Q1 ranking further solidify its core position as the premier publication outlet in this field. Following closely were Parkinsonism & Related Disorders (156 articles), Frontiers in Aging Neuroscience (89 articles), and the Journal of Parkinson’s Disease (65 articles). These journals collectively constitute the primary publication venues for PD-MCI research, predominantly falling within the domains of neuroscience, geriatric neurology, and specialized Parkinson’s disease research.

**TABLE 3:** The top 10 journals in the field of PD-MCI in terms of the number of publications and the top 10 journals in terms of co-citation frequency were identified.

Rank	Journal	Documents	Citations	JCR	Impact factor (2024)	Co-cited journal	Citations	JCR	Impact factor (2024)
1	Movement Disorders	**192**	**18108**	1	7.4	Movement Disorders	**16389**	1	7.4
2	Parkinsonism & Related Disorders	156	5354	2	3.1	Neurology	**13803**	1	9
3	Frontiers in Aging Neuroscience	89	2878	2	4.1	Brain	7123	1	11.2
984	Journal of Parkinson’s Disease	65	1603	2	4	Journal of Neurology, Neurosurgery & Psychiatry	5462	1	9.2
5	Frontiers in Neurology	63	1461	3	2.7	Parkinsonism & Related Disorders	5157	2	3.1
6	Journal of Neurology	59	1901	1	4.8	NeuroImage	3908	1	4.4
7	PLOS ONE	59	2798	1	2.9	Annals of Neurology	3746	1	9.1
8	Journal of Neural Transmission	55	1143	2	3.2	JAMA Neurology	3688	1	21.6
9	Journal of Alzheimer’s Disease	51	1866	2	3.4	Neurobiology of Aging	3416	2	4
10	International Journal of Molecular Sciences	50	1930	1	4.9	The Lancet Neurology	3303	1	48

Values in bold indicate the highest or significantly higher values within the Top 10 for that metric.

In the analysis of cited journals, Movement Disorders also ranked first (16,389 co-citations), indicating that it serves not only as a principal publishing platform but also as the most frequently cited knowledge source by researchers in the field, forming a robust intra-domain citation cycle. However, what is more striking is the high co-citation frequency of a series of high-impact, top-tier neuroscience and clinical neurology journals, such as Neurology, Brain, Journal of Neurology, Neurosurgery & Psychiatry, Annals of Neurology, JAMA Neurology, and The Lancet Neurology. Although these journals may not necessarily publish the highest number of PD-MCI articles themselves, they are frequently co-cited by researchers in the field. This clearly demonstrates that while PD-MCI research is concentrated in specialized journals, its theoretical foundations and methodological approaches are profoundly influenced by mainstream literature in broader, high-impact neuroscience and clinical neurology. Foundational research and major discoveries published in these leading journals constitute an indispensable knowledge cornerstone for the field. Furthermore, the high co-citation rates of NeuroImage and Neurobiology of Aging reflect the significance of neuroimaging and aging neurobiology in PD-MCI research. Taken together, PD-MCI research has established an academic ecosystem characterized by specialized journals serving as core publication vehicles, while concurrently drawing extensively upon the knowledge resources of top-tier neuroscience journals.

Figure 5B systematically presents the disciplinary distribution characteristics and knowledge integration trends of PD-MCI research through a network of multicolored dot clusters and interdisciplinary connecting pathways. The left quadrant of the figure concentrates applied disciplines such as pharmacology and clinical medicine, while the right quadrant features methodological disciplines like mathematical modeling and systems science. The central region forms a cross-domain bridge through molecular biology and neuroscience. Wavy connecting lines reveal knowledge permeation from research on neurodegenerative mechanisms into non-traditional areas such as computational modeling and ecotoxicology. In particular, the strong connection between systems biology and clinical medicine highlights a paradigm shift in PD-MCI research from singular pathological analysis toward multi-omics integration. This disciplinary topological structure confirms the interdisciplinary nature of PD-MCI as a complex disease, providing visual evidence for bibliometric research and indicating that the field is evolving into a global research landscape centered on neuroscience with synergistic innovation across multiple methodologies.

### Analysis of co-cited references

Co-citation analysis identifies foundational works that are frequently cited together in subsequent research. Table 4 lists the top 10 co-cited references, primarily comprising guidelines and articles. The guideline “Diagnostic Criteria for Mild Cognitive Impairment in Parkinson’s Disease: Movement Disorder Society Task Force Guidelines” (Litvan et al., 2012), established by the International Parkinson and Movement Disorder Society (MDS), delineates diagnostic criteria for PD-MCI. It proposes a two-level assessment system based on clinical observation and neuropsychological testing, requiring abnormalities in at least two cognitive domain tests to define single/multi-domain subtypes, while excluding cognitive impairment attributable to other etiologies. The guidelines emphasize that cognitive function should not significantly impair daily independence. These criteria aim to unify the identification framework for PD-MCI, support early screening of patients at high risk for dementia, facilitate clinical interventions, and provide a foundation for research into cognitive decline mechanisms and biomarker validation. Future efforts should focus on refining the reliability of these standards through long-term studies and exploring their association with pathological progression.

**TABLE 4:** PD-MCI studies were cited as the top 10 articles.

Co-cited reference	citations	Journal	Types
Diagnostic criteria for mild cognitive impairment in Parkinson’s disease: Movement Disorder Society Task Force guidelines. Mov Disord. 2012 Mar;27(3):349-56. doi: 10.1002/mds.24893. Epub 2012 Jan 24. PMID:22275317; PMCID: PMC3641655.	956	Movement Disorders	Guidelines
Clinical diagnostic criteria for dementia associated with Parkinson’s disease. Mov Disord. 2007 Sep 15;22(12):1689-707; quiz 1837. doi: 10.1002/mds.21507. PMID:17542011.	605	Movement Disorders	Guidelines
“Mini-mental state.” A practical method for grading the cognitive state of patients for the clinician. J Psychiatr Res. 1975 Nov;12(3):189-98. doi: 10.1016/0022-3956(75)90026-6. PMID:1202204.	480	Journal of Psychiatric Research	Review
Accuracy of clinical diagnosis of idiopathic Parkinson’s disease: a clinico-pathological study of 100 cases. J Neurol Neurosurg Psychiatry. 1992 Mar;55(3):181-4. doi: 10.1136/jnnp.55.3.181. PMID:1564476; PMCID: PMC1014720.	472	Journal of Neurology, Neurosurgery & Psychiatry	Article
The Montreal Cognitive Assessment, MoCA: a brief screening tool for mild cognitive impairment. J Am Geriatr Soc. 2005 Apr;53(4):695-9. doi: 10.1111/j.1532-5415.2005.53221.x. Erratum in: J Am Geriatr Soc. 2019 Sep;67(9):1991. doi: 10.1111/jgs.15925. PMID:15817019.	376	Journal of the American Geriatrics Society	Guidelines
Parkinsonism: onset, progression and mortality. Neurology. 1967 May;17(5):427-42. doi: 10.1212/wnl.17.5.427. PMID:6067254.	341	Neurology	Review
Staging of brain pathology related to sporadic Parkinson’s disease. Neurobiol Aging. 2003 Mar-Apr;24(2):197-211. doi: 10.1016/s0197-4580(02)00065-9. PMID:12498954.	317	Neurobiol Aging	Article
The Sydney multicenter study of Parkinson’s disease: the inevitability of dementia at 20 years. Mov Disord. 2008 Apr 30;23(6):837-44. doi: 10.1002/mds.21956. PMID:18307261.	317	Movement Disorders	Article
Systematic review of levodopa dose equivalency reporting in Parkinson’s disease. Mov Disord. 2010 Nov 15;25(15):2649-53. doi: 10.1002/mds.23429. PMID:21069833.	302	Movement Disorders	Review
MDS Task Force on mild cognitive impairment in Parkinson’s disease: critical review of PD-MCI. Mov Disord. 2011 Aug 15;26(10):1814-24. doi: 10.1002/mds.23823. Epub 2011 Jun 9. PMID:21661055; PMCID: PMC3181006.	279	Movement Disorders	Review

**FIGURE 6:**
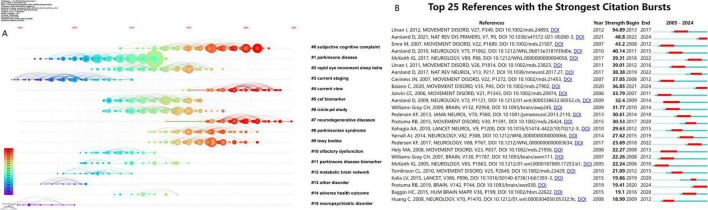
**(A)** The timeline view of the co-cited references network. **(B)** The top 25 references with the strongest citation bursts.

As shown in Figure 6A, the timeline clustering of co-cited references indicates that the nodes on the same horizontal line form a cluster. The labels on the right indicate the theme of each cluster, and the size of the nodes is positively correlated with the frequency of co-citation. The time progresses from left to right. Recent research hotspots include “#0 subjective cognitive complaint,” “#4 current view,” “#7 neurodegenerative diseases,” “#8 parkinsonian syndrome,” and “#9 lewy bodies.”

As shown in Figure 6B, the burst analysis of co-cited references visually presents the duration of research hotspots. The timeline spans from 2005 to 2024, with red bars indicating the period during which a reference experienced a citation burst. Notably, the article by Litvan et al. (2012) authored in 2012 exhibited the highest burst strength (94.89), active between 2013 and 2017. Furthermore, other references demonstrating significant citation bursts include the work by Aarsland D., “Parkinson disease-associated cognitive impairment” (burst 2022–2024, strength 48.8), and the study by Emre M., “Clinical diagnostic criteria for dementia associated with Parkinson’s disease” (burst 2008–2012, strength 43.2).

### Analysis of keywords

**FIGURE 7:**
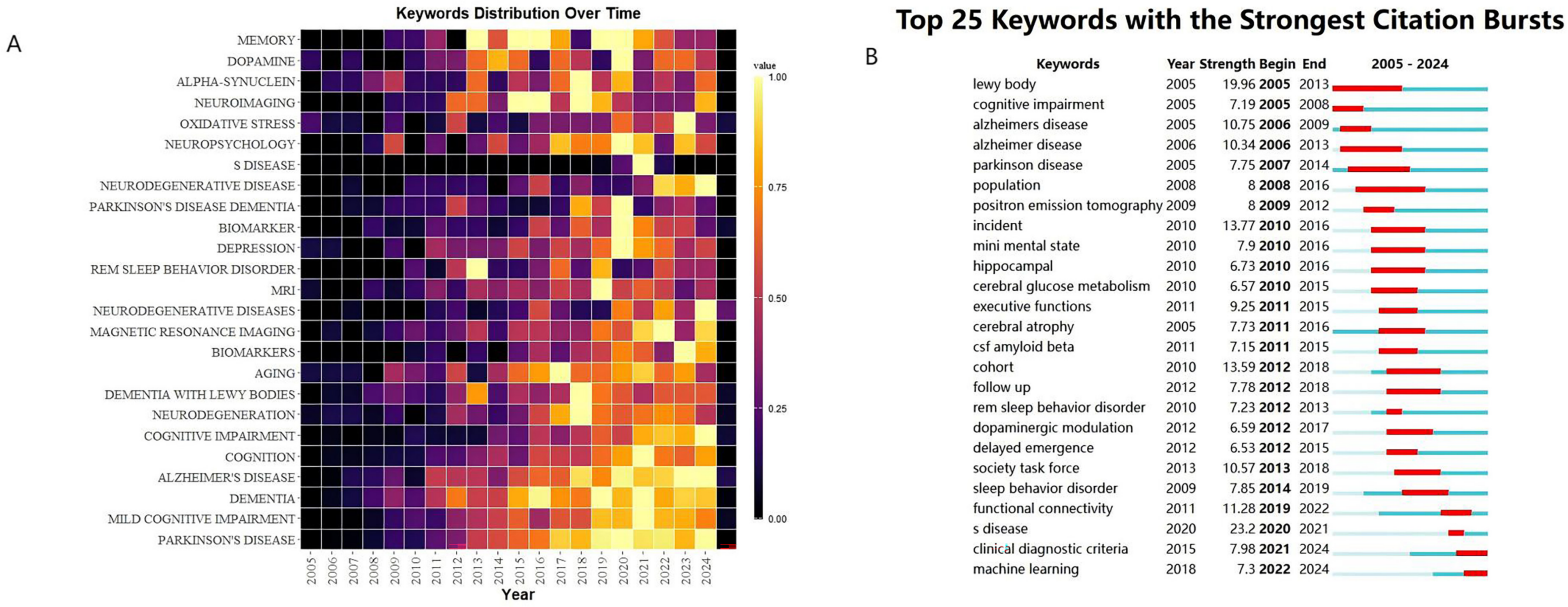
**(A)** The yearly occurrences of top 25 keywords. **(B)** Top 25 keywords with the strongest citation bursts.

As shown in Figure 7A, the keyword annual distribution heatmap system presents the disciplinary evolution path of the PD-MCI field over the past 20 years. Between 2005 and 2015, research focused on molecular pathological mechanisms such as α-synuclein and the dopaminergic system, with studies investigating the association between oxidative stress and neurodegeneration being predominant. After 2015, neuroimaging techniques, biomarker validation, and the classification of PD-MCI subtypes emerged as core growth poles, reflecting a systematic paradigm shift in research from foundational mechanisms toward multimodal diagnostic technologies. The diagonal distribution within the heatmap reveals temporal evolutionary patterns: early high-frequency terms were gradually replaced by PD-MCI-specific indicators. Furthermore, post-2018, neuropsychological assessment tools and quantitative magnetic resonance parameters formed co-occurrence clusters, suggesting the maturation of a clinical-imaging joint diagnostic framework. This evolutionary trajectory highlights that PD-MCI research has entered a phase focused on constructing pathological-clinical correlation models. However, the sustained high frequency of Alzheimer’s disease-related terms also indicates that cross-disease mechanistic comparisons remain a future direction for breakthroughs.

As shown in Figure 7B, the time analysis based on the intensity of keyword bursts from 2005 to 2024 reveals that the PD-MCI research exhibits a three-stage evolutionary characteristic. The early stage (2005–2010) was primarily characterized by exploration of cross-disease mechanisms; the co-occurrence of “Alzheimer disease” (burst strength 10.75) and “Lewy body” (burst strength 19.96) underscores the interdisciplinary research between PD-MCI and the pathologies of Alzheimer’s disease and Lewy body disease, reflecting academic attention to the hypothesis of synergistic pathogenesis involving α-synuclein and β-amyloid. The intermediate stage (2011–2016) shifted toward clinical translational research, with neuroimaging techniques (“positron emission tomography,” strength 8), metabolic biomarkers (“cerebral glucose metabolism,” strength 6.57), and cohort validation (“cohort,” strength 13.59) forming a burst cluster, marking a paradigm shift in PD-MCI diagnosis from symptom description to support by objective biomarkers. The recent stage (2017–2024) has focused on the integration of non-motor symptoms, exemplified by the strong association between “REM sleep behavior disorder” (burst strength 23.0) and dopaminergic modulation (“dopaminergic modulation,” strength 6.59). This reveals research breakthroughs concerning REM sleep behavior disorder as a prodromal marker for PD-MCI, while “society task force” (burst in 2013) corresponds to the development and dissemination of diagnostic criteria by the International Parkinson and MDS. This evolutionary path demonstrates that PD-MCI research has transitioned from basic pathological comparisons to the construction of multimodal diagnostic frameworks, with the biological associations of non-motor symptoms currently representing a core growth area.

Table 5 presents the top 10 keywords based on frequency and centrality, revealing key foci in the research domain. “Mild cognitive impairment” tops the list with a frequency of 1940, underscoring its pivotal role as a core research target. “Parkinson’s disease” and “Alzheimer’s disease” rank high, showing cognitive impairment research often intersects with neurodegenerative conditions. Terms like “dementia,” “diagnostic criteria,” and “diagnosis” highlight clinical emphasis on identification and classification. “Cognitive impairment” reflects broader interest in cognitive - related pathologies, while “lewy body” points to pathological mechanisms. “Dysfunction” at rank 10, with notable centrality, hints at emerging attention to functional deficits. Collectively, these keywords map a landscape spanning cognitive disorder phenotypes, associated neurodegenerative diseases, clinical diagnostics, and pathological explorations.

**TABLE 5:** Top 10 keywords in terms of frequency and centrality.

Rank	Keywords	Frequency	Centrality
1	Mild cognitive impairment	1940	0.01
2	Parkinson’s disease	1838	0
3	Alzheimer’s disease	1190	0.01
4	Dementia	1071	0.01
5	Diagnostic criteria	514	0.01
6	Impairment	362	0.01
7	Cognitive impairment	335	0.02
8	Lewy body	303	0.03
9	Diagnosis	251	0.02
10	Dysfunction	247	0.04

## Discussion

This bibliometric analysis reveals a remarkable and sustained growth trajectory in PD-MCI research over the past two decades, evidenced by a more than twelve-fold increase in annual publications and exponential growth in citation counts. This trajectory signifies PD-MCI’s evolution from a peripheral concern to a central focus within neurodegenerative disease research, likely driven by the imperative for early intervention amidst global aging populations. Notably, the fact that citation growth significantly outpaced publication growth, especially post-2015, underscores the pivotal and increasingly influential role of recent research in shaping the field. Furthermore, our analysis of keyword bursts and co-occurrence clusters delineates a clear paradigm shift: early investigations were predominantly centered on molecular mechanisms, while the period after 2015 witnessed a decisive turn toward clinical translation, characterized by the emergence of neuroimaging biomarkers, PD-MCI subtype classification, and the biological underpinnings of non-motor symptoms. This evolution reflects a maturation of the field toward developing integrated, multimodal diagnostic frameworks that move beyond purely descriptive phenomenology.

Country/region analysis revealed significant disparities in research resource allocation. The United States occupied a leading position with an absolute advantage of 922 publications and 64,138 citations. The centrality of its institutional collaboration network highlights its pivotal role in fostering interdisciplinary synergistic innovation. European countries and Australia, by virtue of high average citations per publication and dense collaborative networks, emerged as core production areas for high-quality research. Although China ranked third with 404 publications, its average citations per publication and network centrality were relatively lagging. This phenomenon of high productivity coupled with lower relative citation impact is not uncommon in rapidly expanding research systems and may be attributable to several factors: the relatively recent surge in PD-MCI research output from China, meaning many publications are still accumulating citations; a higher proportion of publications potentially appearing in regional or national journals with lower international visibility compared to global high-impact journals; differences in collaboration patterns, potentially with stronger domestic networks and less integration into established international high-impact consortia; and variations in research assessment systems that may prioritize quantity in certain contexts. Enhancing the innovativeness of research design, fostering deeper integration into international collaborative networks exemplified by institutions like the Mayo Clinic, and strategically targeting high-impact publication venues are crucial steps for strengthening China’s international academic voice and impact in the PD-MCI field. Suggesting a need to enhance the innovativeness of research design and strengthen its international academic voice. It is noteworthy that institutions such as the Mayo Clinic, through transatlantic collaborations, have successfully bridged neuropathology and clinical cohort research clusters, providing crucial hubs for translational medicine research in PD-MCI.

Abnormal α-synuclein deposition is considered a core driver of neurodegeneration, mediating early cognitive impairment by disrupting synaptic plasticity and neurotransmission (Blommer et al., 2023; Manchinu et al., 2024), while coexisting Alzheimer’s disease-related pathology further accelerates cognitive decline (Liu et al., 2023). Investigations into abnormal neural network connectivity have revealed that reduced functional connectivity within the Default Mode Network (DMN) and fronto-striatal circuits (Delgado-Alvarado et al., 2023; Pan et al., 2024), along with white matter microstructural damage (Zhang et al., 2023; Gao et al., 2024), have emerged as core imaging features of PD-MCI, often preceding the onset of clinical symptoms. The interplay between neuroinflammation and metabolic dysregulation (Lado et al., 2024) offers a novel mechanistic perspective on cognitive decline. In the diagnostic realm, multimodal frameworks integrating structural MRI, functional imaging, and fluid biomarkers have significantly enhanced the precision of subtype classification. Furthermore, the fusion analysis of clinical, imaging, and genetic data using machine learning algorithms (Dadar et al., 2022; Pourzinal et al., 2022; Yang et al., 2023) has driven the identification of heterogeneous subtypes, such as “attentional” and “executive” subtypes, thereby laying the groundwork for personalized interventions (Wang et al., 2022; Garon et al., 2024).

Beyond motor symptoms and core pathological proteins, non-motor symptoms (NMS) and genetic biomarkers are increasingly recognized as critical factors influencing PD-MCI risk, phenotype, and progression. Recent evidence highlights the strong association between specific NMS, such as REM sleep behavior disorder (RBD), and PD-MCI development, suggesting shared underlying neural substrates or prodromal states (Chiang et al., 2024). Furthermore, individuals with PD-MCI experience multifaceted functional impairments linked to NMS burden, impacting daily living and quality of life (Chen et al., 2022). Importantly, genetic factors, particularly GBA variants, have been shown to not only elevate PD-MCI risk but also modulate the effects of cognitive reserve (Chang et al., 2024; Yuan et al., 2025). This underscores the critical need for precision stratification approaches in both research and clinical management of PD-MCI. The foundational diagnostic criteria established by Litvan et al. (2012) and their subsequent international validation by the MDS study group (Geurtsen et al., 2014) remain paramount. However, integrating assessments of key NMS and incorporating genetic risk profiling alongside established cognitive and imaging biomarkers will be essential for developing more comprehensive and personalized diagnostic and prognostic frameworks for PD-MCI.

The first major challenge of diagnostic heterogeneity outlined in the Introduction is clearly reflected in our bibliometric findings. The MDS diagnostic criteria (Litvan et al., 2012) emerged as the most co-cited reference, confirming its pivotal role in standardizing PD-MCI identification. However, our analysis also reveals persistent limitations: variations in cognitive domain cut-off thresholds (Liepelt-Scarfone et al., 2021) and culturally unvalidated assessment tools (Wang et al., 2025) continue to compromise inter-study comparability. Biomarkers like serum cystatin C show promise (Yang et al., 2021; Wang et al., 2025) but require large cohort validation for sensitivity/specificity. Critically, the co-occurrence of multiple pathologies (Gonzalez-Latapi et al., 2021; Carceles-Cordon et al., 2023) revealed in keyword clusters drives heterogeneity in brain structural damage patterns (Cicero et al., 2022), evidenced by DKI-detected white matter microstructural differences between PD-MCI and PDD. This complexity necessitates unified subtype criteria integrating imaging, fluid biomarkers, and non-motor symptom profiles.

Our results highlight significant gaps in addressing the second challenge: developing predictive models that systematically integrate gene-environment interactions. While longitudinal studies exist (Filippi et al., 2020; Puig-Davi et al., 2024), current models fail to incorporate genetic modifiers and their interaction with cognitive reserve (Chang et al., 2024). This is critical as genetic factors accelerate decline trajectories - our keyword bursts revealed “genetic risk” as an emerging frontier. Machine learning applications remain limited by insufficient multimodal data fusion. Future models must integrate dynamic cognitive trajectories with genetic profiles and neuroimaging biomarkers for accurate conversion prediction.

Pharmacological interventions face the third challenge of limited efficacy and mechanistic validation. Cholinesterase inhibitors provide only symptomatic relief with domain-specific response uncertainties (Sun and Armstrong, 2021). Clinical trial designs often overlook subtype heterogeneity (Bayram et al., 2023), while animal models poorly replicate human cognitive diversity (Zhang et al., 2021). Translational bottlenecks are compounded by inadequate algorithms for multimodal data fusion. These limitations are reflected in our results through minimal burst activity in therapeutic keywords.

Converging solutions for these challenges lie in precision medicine frameworks that encompass stratified diagnosis by combining fluid biomarkers with multimodal neuroimaging and AI to detect co-pathologies (Garon et al., 2021), subtype-specific management through defining executive/memory/mixed subtypes via neuropsychology, genetic profiling (Thaler et al., 2024), and dynamic monitoring, and targeted interventions by developing mitochondrial/neuroinflammation therapies (You et al., 2024), optimizing neuromodulation (Han et al., 2021), and implementing Aβ-stratified drug protocols (Bayram et al., 2023). Future research must validate biomarkers through liquid biopsy-neuroimaging-AI integration (Luo et al., 2024) to enable pathology-targeted interventions.

## Limitations

Several limitations of this study should be acknowledged. First, the analysis was restricted to data from the Web of Science Core Collection (WoSCC) to ensure software compatibility, potentially excluding relevant studies indexed in other databases such as PubMed, Google Scholar, and Embase. Second, emerging high-quality publications may not have received sufficient representation due to lower citation frequencies characteristic of recently published works. Finally, the exclusive inclusion of English-language literature may introduce selection bias, particularly regarding region-specific research contributions. These constraints should inform cautious interpretation of the findings while highlighting opportunities for methodological refinement in future bibliometric investigations.

## Conclusion

This study reveals that research on PD-MCI has undergone a three-stage paradigm shift, progressing from the exploration of molecular mechanisms and validation of imaging biomarkers to multi-omics integration. Driven by international collaboration, the United States and Europe have spearheaded core breakthroughs, whereas emerging research systems such as China show potential to enhance impact through cross-regional collaboration and translational research. Current challenges primarily involve the heterogeneity of diagnostic criteria, the incompletely understood co-pathological mechanisms of α-synuclein/Aβ, and the scarcity of disease-modifying therapies. Future efforts should focus on multimodal biomarkers, the development of gene-environment interaction models, and precise interventions targeting the mitochondria-neuroinflammation axis. Such advancements, achieved through interdisciplinary integration, are crucial for enabling the early warning and personalized treatment of PD-MCI.

## Data Availability

The original contributions presented in this study are included in this article/Supplementary material, further inquiries can be directed to the corresponding author.
