# Supplementary material for: Mapping the research landscape of Mild Cognitive Impairment in Parkinson’s disease: a bibliometric and visualization analysis
Source: Front Aging Neurosci. 2025 Sep 10;17:1624420. doi: 10.3389/fnagi.2025.1624420 (PMC12457374; doi:10.3389/fnagi.2025.1624420)
Supplement: Supplementary file 1 [file Data_Sheet_1.pdf]

Entry Terms:

Idiopathic Parkinson Disease

Idiopathic Parkinson's Disease

Lewy Body Parkinson Disease

Lewy Body Parkinson's Disease

Paralysis Agitans

Parkinson Disease, Idiopathic

Parkinson's Disease

Parkinson's Disease, Idiopathic

Parkinson's Disease, Lewy Body

Primary Parkinsonism

Parkinsonism, Primary

Entry Terms:

Mild Cognitive Impairment

Cognitive Impairment, Mild

Cognitive Impairments, Mild

Impairment, Mild Cognitive

Impairments, Mild Cognitive

Mild Cognitive Impairments

Searches:

1: ((((((((((TS=(Idiopathic Parkinson Disease)) OR TS=(Idiopathic Parkinson's Disease)) OR TS=(Lewy Body Parkinson Disease)) OR TS=(Lewy Body Parkinson's Disease)) OR TS=(Paralysis Agitans)) OR TS=(Parkinson Disease, Idiopathic)) OR TS=(Parkinson's Disease)) OR TS=(Parkinson's Disease, Idiopathic)) OR TS=(Parkinson's Disease, Lewy Body)) OR TS=(Primary Parkinsonism)) OR TS=(Parkinsonism, Primary) Editions: WOS.SCI Date Run: Mon Apr 21 2025 23:40:36

GMT+0800 (中国标准时间) Results: 149555

2: (((((TS=(Mild Cognitive Impairment)) OR TS=(Cognitive Impairment, Mild)) OR TS=(Cognitive Impairments, Mild)) OR TS=(Impairment, Mild Cognitive)) OR TS=(Impairments, Mild Cognitive)) OR TS=(Mild Cognitive Impairments) Editions: WOS.SCI Date Run: Mon Apr 21 2025 23:41:21 GMT+0800 (中国标准时间) Results: 54047

3: #2 AND #1 Editions: WOS.SCI Date Run: Mon Apr 21 2025 23:41:27 GMT+0800 (中国标准时间) Results: 3747

4: #2 AND #1 and 1985 or 1979 or 1997 or 1999 or 1993 or 1989 or 1998 or 1995 or 2000 or 1992 or 1990 or 1996 or 2001 or 2004 or 2002 or 2003 or 2025 (Exclude – Publication Years) Editions: WOS.SCI Date Run: Mon Apr 21 2025 23:42:19 GMT+0800 (中国标准时间) Results: 3586

5: #2 AND #1 and 1985 or 1979 or 1997 or 1999 or 1993 or 1989 or 1998 or 1995 or 2000 or 1992 or 1990 or 1996 or 2001 or 2004 or 2002 or 2003 or 2025 (Exclude – Publication Years) and Meeting Abstract or Proceeding Paper or Editorial Material (Exclude – Document Types) Editions: WOS.SCI Date Run: Mon Apr 21 2025 23:43:19 GMT+0800 (中国标准时间) Results: 3278

6: #2 AND #1 and 1985 or 1979 or 1997 or 1999 or 1993 or 1989 or 1998 or 1995 or 2000 or 1992 or 1990 or 1996 or 2001 or 2004 or 2002 or 2003 or 2025 (Exclude – Publication Years) and Meeting Abstract or Proceeding Paper or Editorial Material (Exclude – Document Types) and Book Chapters or Letter or Early Access (Exclude – Document Types) Editions: WOS.SCI Date Run: Mon Apr 21 2025 23:43:22 GMT+0800 (中国标准时间) Results: 3225

7: #2 AND #1 and 1985 or 1979 or 1997 or 1999 or 1993 or 1989 or 1998 or 1995 or 2000 or 1992 or 1990 or 1996 or 2001 or 2004 or 2002 or 2003 or 2025 (Exclude – Publication Years) and Meeting Abstract or Proceeding Paper or Editorial Material (Exclude – Document Types) and Book Chapters or Letter or Early Access (Exclude – Document Types) and Correction or News Item or Data Paper (Exclude – Document Types) Editions: WOS.SCI Date Run: Mon Apr 21 2025 23:43:28 GMT+0800 (中国标准时间) Results: 3219

8: #2 AND #1 and 1985 or 1979 or 1997 or 1999 or 1993 or 1989 or 1998 or 1995 or 2000 or 1992 or 1990 or 1996 or 2001 or 2004 or 2002 or 2003 or 2025 (Exclude – Publication Years) and Meeting Abstract or Proceeding Paper or Editorial Material (Exclude – Document Types) and Book Chapters or Letter or Early Access (Exclude – Document Types) and Correction or News Item or Data Paper (Exclude – Document Types) and Retracted Publication (Exclude – Document Types) Editions: WOS.SCI Date Run: Mon Apr 21 2025 23:43:31 GMT+0800 (中国标准时间) Results: 3218

9: #2 AND #1 and 1985 or 1979 or 1997 or 1999 or 1993 or 1989 or 1998 or 1995 or 2000 or 1992 or 1990 or 1996 or 2001 or 2004 or 2002 or 2003 or 2025 (Exclude – Publication Years) and

Meeting Abstract or Proceeding Paper or Editorial Material (Exclude – Document Types) and Book Chapters or Letter or Early Access (Exclude – Document Types) and Correction or News Item or Data Paper (Exclude – Document Types) and Retracted Publication (Exclude – Document Types) and German or Spanish or Czech or French (Exclude – Languages) Editions: WOS.SCI

Date Run: Mon Apr 21 2025 23:44:28 GMT+0800 (中国标准时间) Results: 3178

10: #2 AND #1 and 1985 or 1979 or 1997 or 1999 or 1993 or 1989 or 1998 or 1995 or 2000 or 1992 or 1990 or 1996 or 2001 or 2004 or 2002 or 2003 or 2025 (Exclude – Publication Years) and Meeting Abstract or Proceeding Paper or Editorial Material (Exclude – Document Types) and Book Chapters or Letter or Early Access (Exclude – Document Types) and Correction or News Item or Data Paper (Exclude – Document Types) and Retracted Publication (Exclude – Document Types) and German or Spanish or Czech or French (Exclude – Languages) and Russian or Turkish or Hungarian or Icelandic (Exclude – Languages) Editions: WOS.SCI Date Run: Mon Apr 21 2025 23:44:34 GMT+0800 (中国标准时间) Results: 3170

11: #2 AND #1 and 1985 or 1979 or 1997 or 1999 or 1993 or 1989 or 1998 or 1995 or 2000 or 1992 or 1990 or 1996 or 2001 or 2004 or 2002 or 2003 or 2025 (Exclude – Publication Years) and Meeting Abstract or Proceeding Paper or Editorial Material (Exclude – Document Types) and Book Chapters or Letter or Early Access (Exclude – Document Types) and Correction or News Item or Data Paper (Exclude – Document Types) and Retracted Publication (Exclude – Document Types) and German or Spanish or Czech or French (Exclude – Languages) and Russian or Turkish or Hungarian or Icelandic (Exclude – Languages) and Polish (Exclude – Languages) Editions: WOS.SCI Date Run: Mon Apr 21 2025 23:44:40 GMT+0800 (中国标准时间)

Results: 3169
